# The Role of the Graphene Oxide (GO) and Reduced Graphene Oxide (RGO) Intermediate Layer in CZTSSe Thin-Film Solar Cells

**DOI:** 10.3390/ma15103419

**Published:** 2022-05-10

**Authors:** Woo-Lim Jeong, Sang-Hyuk Park, Young-Dahl Jho, Soo-Kyung Joo, Dong-Seon Lee

**Affiliations:** School of Electrical Engineering and Computer Science, Gwangju Institute of Science and Technology, 123 Cheomdangwagi-ro, Gwangju 61005, Korea; wljeong@gm.gist.ac.kr (W.-L.J.); psh1118@gist.ac.kr (S.-H.P.); jho@gist.ac.kr (Y.-D.J.)

**Keywords:** Cu_2_ZnSn(S,Se)_4_, thin-film solar cells, graphene oxide, intermediate layer, back contact

## Abstract

Cu_2_ZnSn(S,Se)_4_ (CZTSSe) solar cells with low cost and eco-friendly characteristics are attractive as future sources of electricity generation, but low conversion efficiency remains an issue. To improve conversion efficiency, a method of inserting intermediate layers between the CZTSSe absorber film and the Mo back contact is used to suppress the formation of MoSe_2_ and decomposition of CZTSSe. Among the candidates for the intermediate layer, graphene oxide (GO) and reduced GO have excellent properties, including high-charge mobility and low processing cost. Depending on the type of GO, the solar cell parameters, such as fill factor (FF), were enhanced. Thus, the conversion efficiency of 6.3% was achieved using the chemically reduced GO intermediate layer with significantly improved FF.

## 1. Introduction

Kesterite Cu_2_ZnSn(S,Se)_4_ (CZTSSe) has been continuously studied in recent years to overcome the limitations of Cu(In,Ga)Se_2_ (CIGS), which consumes rare elements. CZTSSe has the advantages of optimal bandgap (1.0–1.5 eV), high absorption coefficient (>10^4^ cm^−1^), and non-toxicity [1,2,3,4,5,6,7,8,9]. However, the power conversion efficiency (PCE) of the CZTSSe solar cells is still lower (13%) than those of competitors such as CIGS (23.35%) [10,11]. This is due to the resistive MoSe_2_ layer formed between the CZTSSe layer and the Mo back contact, the decrease in carrier collection, and the increase in defect states due to the decomposition of CZTSSe. These defect states lead to more Shockley–Read–Hall recombination and degrade device performance. To improve the interface properties of the CZTSSe solar cells, several studies reported intercalating an intermediate layer between the CZTSSe and Mo layers [12,13,14,15,16,17,18,19,20,21]. For example, patterned Al_2_O_3_ [18], carbon [19], Al-doped ZnO [20], CuAlO_2_ [21], etc. have been proposed as intermediate layer materials. Among these, when the Al-doped ZnO layer was inserted into the interface, the decomposition reaction of the Cu_2_ZnSnS_4_ (CZTS), and the formation of the MoS_2_ layer were prevented. However, the excessive thickness of the Al-doped ZnO layer led to the formation of the ZnS secondary phase, resulting in poor performance.

In addition, graphene oxide (GO) is attractive as an intermediate layer material and is particularly favorable for use as an intermediate layer in solar cells due to characteristics such as high-charge mobility (2 × 10^5^ cm^2^ V^−1^ S^−1^), chemical stability, low processing cost, and high transmittance [22,23,24,25,26]. Due to its outstanding properties, it is also used in optoelectronic devices, such as light-emitting diodes, as well as in solar cells [27,28,29]. In the case of the CZTSSe solar cells, for example, Kim et al. conducted measurements to analyze the effect of the GO intermediate layer on the CZTSSe solar cells and reported that it reduces the series resistance and photon loss by preventing MoSe_2_ formation [26].

In this study, we used not only GO but also reduced GO intermediate layers, and analyzed their effects on the CZTSSe solar cells. The reduced GO is more similar to graphene in physical and chemical properties, and is promising as the intermediate layer. The CZTSSe solar cells fabricated using the GO intermediate layers showed better device performance with the thinner MoSe_2_ layer, depending on the GO deposition conditions. We also analyzed the structural disorder according to the reduction of the GOs.

## 2. Materials and Methods

### 2.1. Graphene Oxide Intermediate Layer

Mo back contact layers of 1-μm thickness were deposited on soda-lime glass (SLG, Taewon Scientific Co, Seoul, Korea) substrate using the DC magnetron sputtering system (RSP-5000, SNTEK, Suwon, Korea). Three types of GO samples were obtained by coating GO on the Mo/SLG substrate under different conditions. GO flakes were obtained through the modified Hummers’ method using KMnO_4_, H_2_O_2_, and H_2_SO_4_ [30]. Next, the GO solution was prepared by mixing 30 mg of GO flakes with 10 mL deionized (DI) water. Using a micropipette and spin coater, the 0.5 mL GO solutions were spun once at 3000 rpm for 20 s on the Mo/SLG substrate and dried in air (GO sample). A chemically reduced GO (CrGO) solution was prepared via the chemical reduction of the GO solution [31]. The process involved adding 5 g of p-Toluenesulfonyl Hydrazide (97%, Sigma-Aldrich, St. Louis, MO, USA) to 30 mL of the GO solution and stirring for 24 h at 60 °C. The obtained solution was then filtered and washed with DI water and N,N-Dimethylformamide (99.8%, Sigma-Aldrich, St. Louis, MO, USA). Subsequently, the filtered wet powder was dispersed in isopropyl alcohol. This CrGO solution was coated on the Mo/SLG substrate five times under the same condition as the GO (CrGO sample). Finally, a thermally reduced GO (TrGO) sample was prepared by subjecting the GO sample to rapid thermal annealing (RTA) at 600 °C for 300 s in an N_2_ atmosphere (TrGO sample).

### 2.2. Fabrication of the CZTSSe Solar Cells

Zn, Sn, and Cu metallic precursors were sequentially deposited on the Mo/SLG substrate with various GO intermediate layers via DC (for Zn and Cu layers) and RF magnetron (for Sn layer) sputtering at 1.1 W/cm^2^ of power. The purity values of the sputtering targets for Mo, Zn, Sn, and Cu were 99.5%, 99.95%, 99.95%, and 99.95%, respectively (Taewon Scientific Co, Seoul, Korea). An Ar atmospheric pressure was maintained at 0.4 Pa during the precursor sputtering. The individual sputtering time were determined as the Zn-rich and Cu-poor compositional ratios of the CZTSSe absorber films. The precursors were heated in a graphite box with 0.5 g Se and 0.02 g SeS_2_ powders using RTA system. The RTA chamber with the graphite box was maintained under vacuum for 15 min and then filled with Ar gas to atmospheric pressure. After that, the samples were heated from room temperature to 300 °C in 1000 s, maintained at 300 °C for 1500 s, heated from 300 to 550 °C in 1000 s, and then maintained at 550 °C for 1100 s. Next, to remove unwanted secondary phases, the CZTSSe absorber films were immersed in a 0.05 M KCN aqueous solution for 5 min and cleaned with DI water. A 70-nm CdS buffer layer was deposited by chemical bath deposition at 80 °C, followed by 50-nm intrinsic ZnO and 350-nm aluminum-doped ZnO layers via RF magnetron sputtering at room temperature and 150 °C, respectively. 50-nm Ni, 500-nm Al electrodes were deposited through a shadow mask with an electron beam evaporator. Finally, a 105-nm-thick MgF_2_ layer was deposited as an anti-reflection coating using electron beam evaporator.

### 2.3. Characterization

Scanning electron microscopy (SEM, S-4700, Hitachi, Tokyo, Japan) and atomic force microscopy (AFM, XE-100, Park Systems, Suwon, Korea) were conducted for the surface analysis of the GO coated Mo back contacts and the cross-sectional analysis of the CZTSSe solar cells. Raman scattering measurements (InVia, Renishaw, Gloucestershire, UK) were performed at a 514-nm laser excitation wavelength. The electrical property of the GO coated Mo back contacts was measured using a 4-point-probe (FPP-RS8, Dasol Eng., Cheongju, South Korea). Energy-dispersive X-ray spectroscopy (EDX, S-4700, Hitachi, Tokyo, Japan) was performed for compositional analysis of the CZTSSe absorber films. External quantum efficiency (EQE, QEX7, PV Measurements, Boulder, CO, USA) measurements were performed to estimate the bandgap energies of the CZTSSe solar cells. The device parameters of the CZTSSe solar cells were measured with a class AAA solar simulator (WXS-155S-L2, Wacom, Tokyo, Japan) under conditions of AM 1.5 G, 100 mW/cm^2^, and 25 °C.

## 3. Results

**Figure 1:**
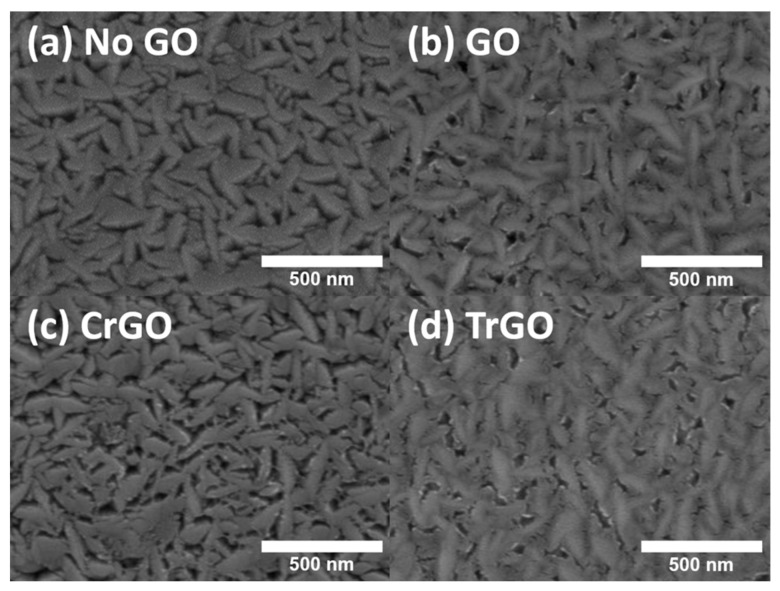
Surface scanning electron microscopy (SEM) images of the Mo back contacts with different intermediate layers: (**a**) no graphene oxide (GO), (**b**) GO, (**c**) chemically reduced GO (CrGO), and (**d**) thermally reduced GO (TrGO).

Figure 1 shows the surface scanning electron microscopy images of the Mo back contacts with different intermediate layers using a GO. If there is no GO, the Mo back contact shows gaps between the hills (Figure 1a). On the other hand, the sample with GO on the Mo back contact had partially filled gaps, as shown in Figure 1b. It occurred because the gap was covered by GO, and the covering ratio would be controlled depending on the concentration of GO solution and the number of deposition cycles. The CrGO sample did not show the covering, which seems to be due to the low concentration of the CrGO solution. The TrGO sample obtained by annealing of GO at 600 °C showed a similar covering ratio, because the same concentration of GO solution was used.

**Figure 2:**
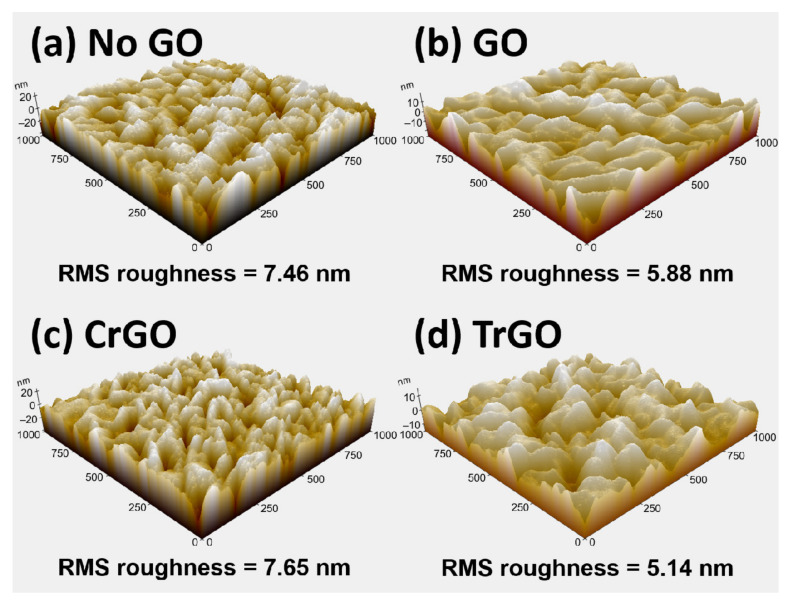
Atomic force microscopy (AFM) images and root-mean-square (RMS) roughness of the Mo back contacts with different intermediate layer: (**a**) no graphene oxide (GO), (**b**) GO, (**c**) chemically reduced GO (CrGO), and (**d**) thermally reduced GO (TrGO).

The results of AFM measurement of the Mo back contacts with various GO intermediate layers are shown in Figure 2. In the absence of the GO, a root-mean-square (RMS) roughness of 7.46 nm was obtained from the Mo layer. On the other hand, the RMS roughness was decreased to 5.88 nm and 5.14 nm when GO and TrGO covered the Mo layer, respectively. As confirmed by the SEM analysis, it is expected that the CrGO was not thick enough to cover the Mo gap due to the low concentration of the CrGO solution. Therefore, the RMS roughness was similar to that of the No GO sample (5.14 nm).

**Figure 3:**
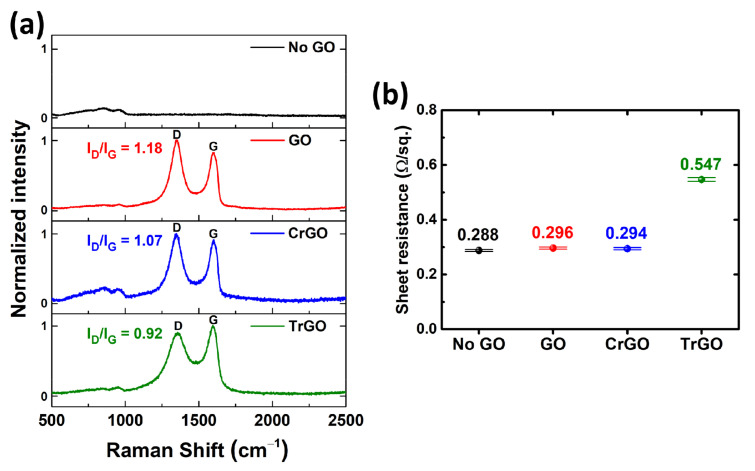
(**a**) Raman spectra and (**b**) sheet resistances of the various graphene oxide intermediate layers on the Mo back contact.

Raman spectroscopy is an effective method for analyzing the structure of carbon nanomaterials. Therefore, a Raman analysis was performed on the GO intermediate layers and the results with the D-band intensity (I_D_)/ G-band intensity (I_G_) ratio are shown in Figure 3a. Except for the No GO sample, all three GO samples showed D and G band at 1351 cm^−1^ and 1600 cm^−1^. The G band is related to the in-plane vibrations of the sp^2^ bond of carbon atoms, and the D band is related to the out of plane vibrations associated with structural defects [32,33]. The GO sample, which does not include the reduction process, showed an I_D_/I_G_ ratio of 1.18, while those of CrGO and TrGO samples showed 1.07 and 0.92 decreases, respectively. Thus, it is expected that the structural disorder of the reduced GO samples (CrGO and TrGO) decreased compared to the GO sample. It was confirmed that the GO layer also exists on the Mo back contacts [30,33]. In addition, the result of 4-point-probe measurement for the GO coated Mo back contacts is shown in the Figure 3b. Since GO layer is very thin and partially covered, the sheet resistance would have been contributed by the Mo layer. Note that the TrGO sample shows the highest sheet resistance (0.547 Ω/sq.). Mo has the characteristic that it is easily oxidized, and it is expected that the RTA process at 600 °C deteriorates the Mo layer.

**Figure 4:**
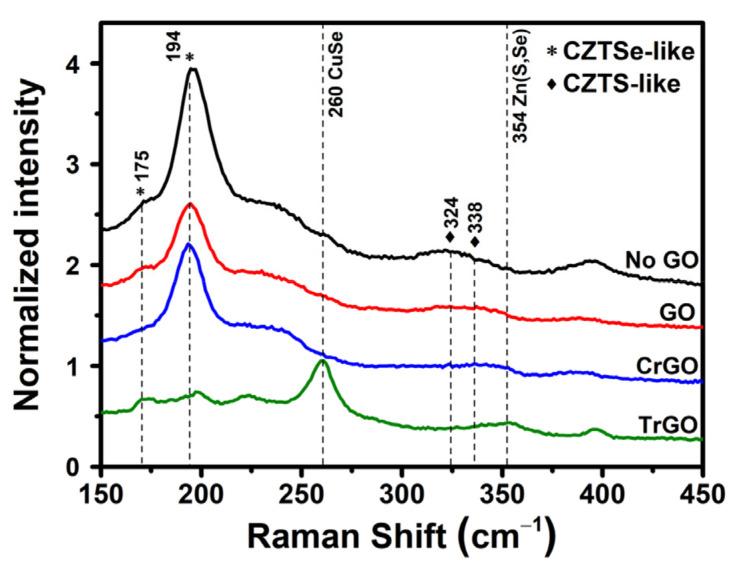
Raman spectra of the CZTSSe absorber films fabricated with the various graphene oxide intermediate layers.

The CZTSSe absorber films were fabricated using various substrates with GO intermediate layers, and their Raman analyses and EDX analyses results are shown in Figure 4 and Table 1. The CZTSSe absorber films mainly showed Cu_2_ZnSnSe_4_ (CZTSe)-like peaks at 175 and 194 cm^−1^ and CZTS-like peaks at 324 and 338 cm^−1^ [34]. The intensities of the CZTSe-like peaks were higher than those of the CZTS-like peaks, which is consistent with the results of EDX analysis that the Se content is higher than the S content. However, the CuSe peak was larger than CZTSSe-related peaks in the TrGO sample, which is assumed to be because the degraded Mo layer prohibits the formation of the CZTSSe. The secondary phase, for instance CuSe, is a conductive shunting path, and the electrical potential difference between the positive and negative electrodes disappears due to the shunting path. Therefore, even if the charge carriers are excited inside the absorber film, the carriers cannot be collected. Furthermore, the GO and CrGO samples showed peak shift compared to the No GO samples, which may be due to incorporation of carbon atoms [35]. The incorporation will change the bonding between the atoms in CZTSSe, resulting in the peak shift.

**Figure 5:**
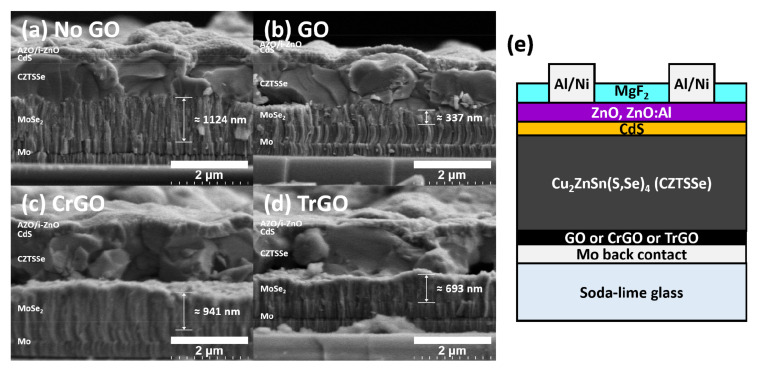
(**a**–**d**) Cross-sectional SEM images and (**e**) schematic of the CZTSSe solar cells fabricated with different graphene oxide intermediate layers.

Figure 5 shows the results of cross-sectional SEM analysis on the completed CZTSSe solar cells. All samples showed about 1-µm thickness of the CZTSSe absorber film because their process condition was the same. On the other hand, the thicknesses of MoSe_2_ layer showed different values due to the various GO intermediate layers, and were obtained as 1124, 337, 941, and 693 nm, respectively. Interestingly, GO samples inhibited the formation of the MoSe_2_ layer more than other samples. As confirmed by the surface SEM analysis in Figure 1, this seems to be due to the high covering ratio of the GO sample. In the case of the CrGO, the effect of the inhibiting the formation of MoSe_2_ layer was insignificant because the concentration of the CrGO solution was lower than that of the GO, and the Mo back contact was hardly covered.

**Figure 6:**
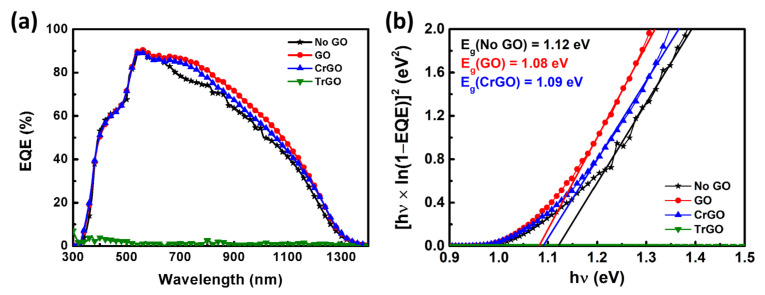
(**a**) External quantum efficiency (EQE) curves of the CZTSSe solar cells and (**b**) the bandgap energies (*E_g_*) of the CZTSSe absorber layers obtained from the EQE data.

To estimate the bandgap energy, EQE measurements were performed on the CZTSS solar cells using the various GO intermediate layers, as show in Figure 6. Both GO and CrGO samples showed more improved EQE than the No GO sample in the 600–1100-nm wavelength range. It seems that the MoSe_2_ layer suppressed by the GO intermediate layer improved carrier collection. In addition, some studies have shown that light absorption and short circuit current (*J_SC_*) are improved due to the mitigation of the interference between incident and reflected light through the intermediate layer [26,36]. The TrGO sample showed little carrier collection, and it seems that the conductive secondary phase acted as shunting paths and prevented carrier collection. The bandgap energies of the No GO, GO, and CrGO samples were 1.12, 1.08, and 1.09 eV, respectively.

**Figure 7:**
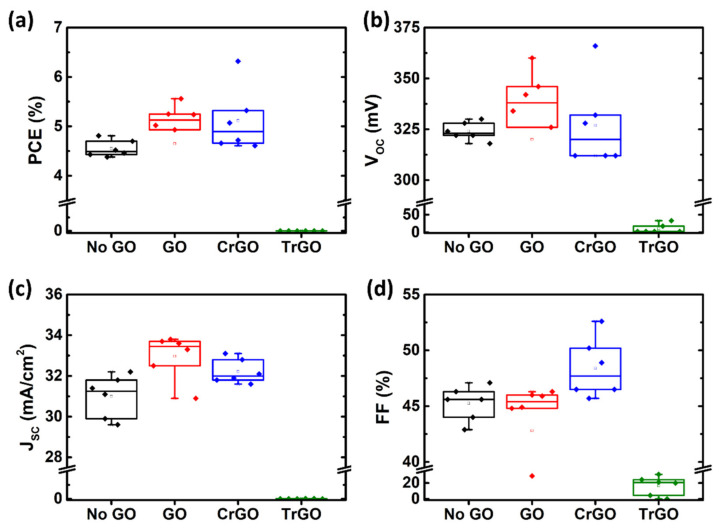
Statistical box charts of (**a**) the power conversion efficiency (PCE), (**b**) open-circuit voltage (*V_OC_*), (**c**) short-circuit current density (*J_SC_*), and (**d**) fill factor (FF) of the CZTSSe solar cells fabricated with the various graphene oxide intermediate layers (*n* = 6).

Figure 7 shows statistical box charts for solar cell parameters obtained from the CZTSSe solar cells fabricated with various GO samples. The GO and CrGO samples showed average PCEs of 4.65% and 5.12%, respectively, which is higher than the No GO sample of 4.55%. From Figure 7b–d, the PCE improvement of the GO sample was mainly contributed by the *V_OC_* and *J_SC_*, while that of the CrGO sample was more significantly contributed by FF. This difference was probably caused by the degree of covering of the GO intermediate layer. When the passivation of the intermediate layer is excessive, the *J_SC_* and *V_OC_* can increase, but the FF can decrease [37]. It is supposed that the FF decreases of the GO sample were due to the high concentration of the GO solution and the relatively high sheet resistance of the GO [38]. The high concentration of the GO solution strongly suppressed MoSe_2_ formation, but the GO layer acted as the electrical resistance. On the other hand, the CrGO sample exhibited a relatively low concentration of GO solution and improved FF, resulting in better performance (Table 2). Both samples using the GO intermediate layer showed improvement in *J_SC_* and *V_OC_*, it is assumed that the *J_SC_* improvement is due to the reduced interference between the incident light and the reflected light, as mentioned in Figure 6. Moreover, the GO intermediate layer may restrain the decomposition of the CZTSSe, reduce the defect state, and lead to the increase in the *V_OC_*.

## 4. Conclusions

We demonstrated the effect of the GO and reduced GO intermediate layers on the CZTSSe solar cells. The GO sample was produced using the modified Hummers’ method, and the CrGO and TrGO samples were prepared through the chemical and thermal reduction of the GO. Prior to the analysis of solar cells, surface analyses were performed on the Mo back contacts coated with various GO intermediate layers, and differences in the covering ratios of the samples were verified via SEM and AFM measurements. In addition, Raman spectroscopy confirmed that the I_D_/I_G_ ratio of GO, CrGO, and TrGO decreased from 1.18 to 0.92 in, depending on the reduction condition. The CZTSSe solar cells fabricated using those GO samples showed relatively thin MoSe_2_ layers and improved carrier collection in the EQE measurements. In particular, the GO sample made with high concentrations of GO solutions showed significant improvements in *V_OC_* and *J_SC_*. However, the FF of the GO sample decreased due to the high electrical resistance of the GO. On the other hand, the CrGO sample showed significant enhancement in FF, owing to the moderate passivation. As a result, the CZTSSe solar cell fabricated using the CrGO intermediate layer showed PCE of 6.3%, *V_OC_* of 0.366 V, and *J_SC_* of 32.8 mA/cm^2^. To the best of our knowledge, this is the first time that the effect of various GO intermediate layers on CZTSSe solar cells have been analyzed. Thus, we believe that this study on the GO intermediate layers made through a facile solution process may accelerate research into applying GO intermediate layers to the CZTSSe solar cell.

## Figures and Tables

**Table 1:** Elemental composition and compositional ratios of the CZTSSe absorber films fabricated with the various graphene oxide intermediate layers.

	Elemental Composition (at %)					Compositional Ratios			
CZTSSe Sample	Cu	Zn	Sn	S	Se	Zn/Sn	Cu/ (Zn + Sn)	Se/S	(Se + S)/ Metal
No GO	20.53	16.79	11.2	10.28	41.21	1.50	0.73	4.01	1.06
GO	21.12	16.25	11.26	7.09	44.28	1.44	0.77	6.25	1.06
CrGO	20.35	16	11.32	10.93	41.4	1.41	0.74	3.79	1.10
TrGO	20.92	15.83	10.67	11.57	41	1.48	0.79	3.54	1.11

**Table 2:** Solar cell parameters obtained from *J-V* curves and external quantum efficiency data of the CZTSSe solar cells with different intermediate layers ^1^.

Sample	PCE (%)	*V_OC_* (V)	*J_SC, J-V_* (mA/cm^2^)	FF (%)	*J_SC, EQE_* (mA/cm^2^)	*E_g_* (eV)
No GO	4.8	0.322	32.2	46.3	31.2	1.12
GO	5.6	0.360	33.6	46.0	33.3	1.08
CrGO	6.3	0.366	32.8	52.6	32.3	1.09
TrGO	0	0.018	0	21.0	0.7	–

^1^ The active area of each cell was 0.3 cm^2^. PCE, power conversion efficiency; *J_SC_*, short-circuit current; *V_OC_*, open-circuit voltage; FF, fill factor; EQE, external quantum efficiency; *E_g_*, bandgap energy.

## Data Availability

Data are contained within the article.
